# Factors Associated with Prolonged Hospitalisation in Women with Breast Cancer: Evidence from a Large German Multicentre Study

**DOI:** 10.3390/clinpract16090169

**Published:** 2026-09-11

**Authors:** Lisa Cordes, Karel Kostev, Matthias Kalder

**Affiliations:** 1 Department of Gynecology and Obstetrics, University Hospital, Philipps-University Marburg, Baldingerstraße, 35043 Marburg, Germany; 2 Epidemiology, IQVIA, Unterschweinstiege 2-14, 60549 Frankfurt am Main, Germany

**Keywords:** breast cancer, hospital length of stay, prolonged hospitalisation, comorbidity, Elixhauser comorbidity categories, perioperative complications, Germany

## Abstract

**Background/Objectives**: Breast cancer is the most common malignancy among women worldwide and poses a significant burden on healthcare systems. Despite evidence that treatment type is associated with hospital length of stay (LOS), current Germany-wide data on this topic are lacking. The present study aims to identify clinical and procedural factors associated with prolonged hospitalisation in women hospitalised for breast cancer in Germany. **Methods**: This multicentre cross-sectional study analysed anonymised inpatient data from 38 German hospitals (IQVIA database), including 16,062 female breast cancer hospitalisation episodes (inpatient cases; patient-level linkage was not possible due to anonymisation, so these may not represent 16,062 distinct women) treated between January 2019 and December 2024. The primary outcome was LOS; prolonged hospitalisation was defined as LOS > 7 days (75th percentile). Comorbidities were assessed using individual comorbidity categories defined according to the Elixhauser classification (retained at ≥1% prevalence). The primary multivariable analysis was restricted to characteristics documented independently of the in-hospital course (age, tumour site, metastatic status, chronic comorbidities). In-hospital complications, procedures, and diagnoses of ambiguous timing were analysed separately as secondary, descriptive associations. LOS was modelled using negative binomial regression (Poisson regression as a sensitivity analysis) and prolonged LOS using logistic regression, both with hospital-clustered robust (sandwich) standard errors. **Results**: The overall median LOS was 4 days (IQR 3–7); 19.3% of hospitalisation episodes were prolonged. In the primary analysis, longer LOS was associated with age > 70 years (RR 1.14; 95% CI 1.08–1.20), distant metastases (RR 1.76; 95% CI 1.51–2.04), lymph node metastases (RR 1.09; 95% CI 1.05–1.12), congestive heart failure (RR 1.44; 95% CI 1.31–1.59), anaemia (RR 1.41; 95% CI 1.14–1.75) and depression (RR 1.14; 95% CI 1.03–1.26). In the secondary descriptive analysis, in-hospital complications showed the strongest associations with longer LOS, particularly postoperative infection (RR 2.28), wound disruption (RR 1.92) and pneumonia (RR 1.45). Breast-conserving surgery and partial breast resection were associated with shorter LOS, while mastectomy, blood transfusions, and complex intensive care were associated with prolonged stays; the small inpatient radiotherapy subgroup (1.9%) most likely reflects a highly selected, predominantly palliative population rather than an association attributable to radiotherapy itself. **Conclusions**: Prolonged hospitalisation was associated with older age, documented lymph node and distant metastases, and chronic somatic comorbidities including congestive heart failure, anaemia, chronic kidney disease and depression, and, in secondary descriptive analyses, with perioperative complications and intensive procedures. The strongest correlates of prolonged stay—in-hospital complications and intensive procedures—are documented during rather than before the hospitalisation and therefore cannot themselves support early risk identification; however, the baseline characteristics documented independently of the in-hospital course (age, metastatic disease, chronic comorbidity burden) may usefully inform structured perioperative management and discharge planning in Germany.

## 1. Introduction

Breast cancer, the malignant degeneration of the mammary gland, medically referred to as mammary carcinoma, is the most common cancer in women and poses a significant global challenge to healthcare systems [1]. According to global cancer statistics, there were more than 2.3 million new cases of breast cancer in 2022, accounting for 11.6% of all newly diagnosed cancers in that year [2]. The incidence is significantly higher in economically developed countries than in lower-income countries [3]. In 2019, 71,375 women in Germany received an initial diagnosis of breast cancer, bringing the age-standardised incidence to 114.6 per 100,000 women [4].

Breast cancer poses a challenge both in terms of health and health economics. In Germany, one in eight women will be affected by malignant degeneration of the mammary gland during their lifetime, which is why it is particularly important to prevent this disease, detect it early, and treat it as effectively as possible in order to avoid complications and enable a cure without recurrence [5]. Surgical treatments in particular carry an inherent risk of complications, which are often predictable or preventable [6]. This is where action can be taken to achieve optimal treatment outcomes and care for patients in hospital for as long as necessary, but no longer than required.

Previous studies investigating the length of hospital stays for breast cancer have mostly been conducted in other countries and no longer contain up-to-date data. However, these authors showed that the type and extent of treatment are key factors associated with the length of stay [7,8]. The choice of surgical treatment, including between breast-conserving therapy, mastectomy with or without reconstruction, and removal of axillary lymph nodes with the need for drainage, has also been reported to be associated with the length of hospital stay [8]. Prolonged hospital stays have likewise been described in the presence of postoperative complications such as wound healing disorders [8]. Patient-related factors such as age or existing comorbidities have also been reported to be relevant. Unfortunately, current and specific data for Germany are not available [8].

This study therefore aims to identify the factors that are associated with prolonged hospital stays in breast cancer hospitalisation episodes in Germany. Beyond the general scarcity of recent German inpatient data, to our knowledge, this is the first multicentre German study to combine granular, Elixhauser-based comorbidity assessment with a detailed breakdown of surgical, systemic, and radiotherapy procedures in relation to LOS in breast cancer, across hospitals of differing care levels.

## 2. Materials and Methods

This multicenter, cross-sectional study was based on anonymized inpatient data provided by IQVIA and included information from 49 hospitals across Germany. The database comprises approximately 2,143,071 hospitalizations documented between 1 January 2019 and December 2024. It represents a broad range of hospital types, including university medical centres, maximum-care and standard-care hospitals, primary care institutions, and specialised facilities.

Participating hospitals routinely transmit their inpatient case data to the Institute for the Hospital Remuneration System (InEK) in accordance with §21 of the German Hospital Compensation Act (KHEntgG). Data submission follows a standardised reporting format. Prior to transmission, all records are fully anonymized in compliance with applicable data protection regulations, with patient- and case-level identifiers removed or replaced.

This data source has previously been used in epidemiological research evaluating hospital length of stay [9,10].

### 2.1. Study Population

The study population comprised 16,062 inpatient cases of women aged ≥ 18 years with a primary diagnosis of breast cancer (ICD-10: C50) documented between January 2019 and December 2024. Only hospitalisations with a length of stay of at least two days were eligible for inclusion. Four episodes were admitted before 1 January 2019 and discharged within the study period; these were retained, so the admission-year variable spans 2018–2024, while the study period itself is 2019–2024. The 16,062 records represent hospitalisation episodes (inpatient cases) rather than necessarily 16,062 distinct women: the anonymisation procedure applied to this dataset removes patient-level identifiers, so repeat admissions by the same woman cannot be identified or excluded, and the unit of analysis throughout this manuscript is the hospitalisation episode.

Breast cancer cases were identified in 38 of the 49 hospitals included in the database. The median number of breast cancer cases treated per hospital was 110 (interquartile range [IQR]: 7–655).

### 2.2. Study Outcome and Variables

The primary outcome was hospital length of stay (LOS), measured in days. In addition to reporting the median LOS, we examined the proportion of hospitalisation episodes with prolonged hospitalisation, defined as LOS > 7 days, corresponding to the 75th percentile of LOS in the overall study population.

Demographic characteristics included mean age and predefined age categories (≤50, 51–60, 61–70, and >70 years).

Breast cancer location was classified according to ICD-10 codes as follows: nipple and areola (C50.0), central portion (C50.1), upper-inner quadrant (C50.2), lower-inner quadrant (C50.3), upper-outer quadrant (C50.4), lower-outer quadrant (C50.5), axillary tail of the breast (C50.6), overlapping sites (C50.8), and unspecified site (C50.9). The presence of lymph node metastases (ICD-10: C77) and distant metastases (ICD-10: C78, C79) was additionally recorded.

Comorbidity was assessed using ICD-10 codes based on the Elixhauser classification [11], selecting individual comorbidity categories rather than a single summary index score. Of the 31 comorbidity categories included in the index, only those documented in at least 1% of the study population were considered, to ensure adequate statistical power for each retained category while excluding categories too rare to estimate reliably in this cohort. These included congestive heart failure (ICD-10: I50, I11, I13), cardiac arrhythmias (I47–I49), hypertension (I10), chronic pulmonary disease (J40–J44, J47), diabetes mellitus (E10–E14), thyroid disorders (E00–E07), chronic kidney disease (N18, N19), obesity (E66), anaemia (D50–D53, D55–D59), fluid and electrolyte disorders (E86, E87), and depression (F32, F33). Present-on-admission flags or diagnosis timestamps were not available in this dataset, so it is not possible to determine with certainty whether a given comorbidity code was recorded before or during the hospitalisation. Rather than assuming that any diagnosis was “known at admission”, we therefore classified all variables a priori, on clinical grounds, into three groups. Group A comprised characteristics documented independently of the in-hospital course (age, tumour site, lymph node and distant metastases). Group B comprised comorbidity categories that are chronic by definition and can be regarded as present before admission with high clinical plausibility (congestive heart failure, hypertension, chronic pulmonary disease, diabetes mellitus, thyroid disorders, chronic kidney disease, obesity, depression). Group C comprised diagnoses whose timing cannot be established in these data because they may arise de novo during an inpatient stay (fluid and electrolyte disorders, cardiac arrhythmias). Group D comprised in-hospital complications and procedures, which by definition are documented during the hospitalisation whose length is the outcome. Groups A and B constitute the primary multivariable model; Groups C and D are reported separately as secondary, descriptive associations. This classification is based on clinical plausibility and not on a present-on-admission flag, and is discussed further under Limitations.

In addition, acute in-hospital complications were evaluated, including sepsis (A40, A41, R57.2), postoperative infections (T81.4), haemorrhage and haematoma (T81.0), wound disruption (T81.3), pneumonia (J12–J18), and renal, liver, or respiratory failure (N17, K72.0, K72.9, J80, J96).

Procedure-related variables were defined according to the German Operation and Procedure Classification System (OPS), based on the German modification of the International Classification of Procedures in Medicine (ICPM). These included chemotherapy (OPS: 8-542, 8-543, 8-544), radiotherapy (OPS: 8-52), breast-conserving surgery (OPS: 5-870.20, 5-870.21, 5-870.60, 5-870.61, 5-870.90, 5-870.91; defined as excision of the tumour only, preserving the remaining breast tissue), partial breast resection (OPS: 5-870.a; defined as removal of a wider tissue segment including a margin of healthy breast tissue), mastectomy (OPS: 5-872, 5-874, 5-877), operations on the lymphatic system (OPS: 5-40), transfusion of blood cells (OPS: 8-800), and complex intensive care treatment (OPS: 8-980, 8-98f, 8-98d). The three surgical categories (breast-conserving surgery, partial breast resection, mastectomy) were coded as mutually exclusive, assigned in the priority order partial resection > mastectomy > breast-conserving surgery where more than one code was present; 30.6% of admissions (4920/16,062) carried none of these three codes.

### 2.3. Statistical Analysis

Associations between clinical variables and hospital length of stay (LOS) were evaluated using multivariable negative binomial regression models. Effect estimates are reported as rate ratios (RRs) with corresponding 95% confidence intervals (CIs) and *p*-values. Associations with prolonged LOS were assessed using multivariable logistic regression models, and results are presented as odds ratios (ORs) with 95% CIs and *p*-values. A count model with a log link was used because LOS is a non-negative count outcome. Poisson regression was fitted first, but the mean-variance assumption, assessed via deviance and Pearson goodness-of-fit statistics, indicated overdispersion (deviance/df = 1.74; Pearson χ^2^/df = 2.08). The negative binomial model showed substantially better fit (deviance/df = 0.86; Pearson χ^2^/df = 1.06; AIC 76,695 vs. 82,481 for the Poisson model) and was therefore used as the primary LOS model, with the Poisson model retained as a sensitivity analysis (Supplementary Materials). Both models were estimated in a generalised estimating equations framework with an independence working correlation structure and empirical (sandwich) standard errors clustered on hospital; this is the estimator referred to throughout as cluster-robust, and it is the same specification described as Poisson GEE in our earlier response letter. Prolonged LOS (>7 days) was defined using the 75th percentile of the LOS distribution; because only 19.3% of the cohort exceeded 7 days, this threshold is data-driven rather than a clinically validated cutoff, and sensitivity analyses using ≥5-day and ≥7-day definitions were additionally performed (Supplementary Materials).

The primary models were adjusted for age, breast cancer site, lymph node and distant metastases, and the chronic comorbidity categories of Groups A and B (the cohort comprises women only, so sex was not an adjustment variable). The secondary models additionally included the diagnoses of ambiguous timing (Group C) and the in-hospital complications and procedures (Group D). Statistical significance was defined as a two-sided *p*-value < 0.05. All analyses were conducted using SAS software, version 9.4 (SAS Institute, Cary, NC, USA). The unspecified site (C50.9) had the longest median LOS of all tumour-site categories; using it as the reference category would make most other sites appear spuriously protective, so the upper-outer quadrant (C50.4, the most frequent well-specified site, 36.5%) was used as the reference category for all tumour-site comparisons reported below. Because the 38 participating hospitals showed substantial heterogeneity in case volume, all multivariable models used cluster-robust (sandwich) standard errors with hospital as the clustering variable, cross-validated against multilevel (random-intercept) models; one hospital alone contributed 3764/16,062 admissions (23.4%), and a sensitivity analysis excluding this hospital is reported in the Supplementary Materials. Additional sensitivity analyses restricted the multivariable model to the 11,142 (69.4%) admissions with a coded surgical procedure, adjusted for admission year (2018–2024) to account for potential COVID-19-related changes in practice; the admission-year variable records the year of admission, which for four transfer episodes precedes 1 January 2019 although the episode itself falls within the study period, and, conversely, added cardiac arrhythmias and fluid/electrolyte disorders—the two comorbidity categories most plausibly subject to onset during rather than before the hospitalisation, and therefore excluded from the primary model—to the comorbidity profile (Supplementary Materials). Given the large number of statistical comparisons, a Benjamini–Hochberg false discovery rate (FDR) correction was applied to the *p*-values of the main multivariable model. This observational study is reported in accordance with the STROBE guideline (Table S9).

## 3. Results

### 3.1. Baseline Characteristics

The study included 16,062 inpatient cases of women with breast cancer. The mean age was 62.8 years (SD 14.0). Approximately one third of episodes involved women older than 70 years (31.8%), while 21.1% involved women aged ≤ 50 years. The median number of comorbidities per case (of the ten retained categories) was 1 (Q1–Q3: 0–1; mean 0.81).

The most frequent tumour location was the upper-outer quadrant (36.5%), followed by overlapping sites (19.3%) and the unspecified site (13.0%). Lymph node metastases (C77) were documented in 21.8% of episodes and distant metastases in 17.2%.

Hypertension (36.9%) and thyroid gland disorders (16.4%) were the most prevalent comorbidities. Acute in-hospital complications were relatively rare, although renal, liver, or respiratory failure occurred in 4.2% and haemorrhage/haematoma in 2.9%. The most common procedures were operations on the lymphatic system (62.2%), partial breast resection (35.3%), and mastectomy (21.3%) (Table 1).

### 3.2. Median Hospital Length of Stay and Prolonged Hospitalisation

The overall median hospital length of stay (LOS) was 4 days (IQR 3–7), and 19.3% of hospitalisation episodes were prolonged (>7 days). Median LOS increased with age and reached 5 days (IQR 3–8) in episodes involving women > 70 years, of which 26.9% were prolonged. Episodes with an unspecified tumour site had a median LOS of 6 days (IQR 3–9) and the highest proportion of prolonged hospitalisation (35.1%). Similarly, episodes with documented distant metastases had a median LOS of 7 days (IQR 4–12), of which 47.3% were prolonged. Substantially longer LOS was observed in episodes with congestive heart failure (median 8 days; 53.6% prolonged hospitalisation), chronic kidney disease (median 7 days; 48.0%), and fluid and electrolyte disorders (median 9 days; 58.3%) (Table 2).

Acute complications were strongly associated with prolonged hospitalisation at the descriptive level. For example, episodes with sepsis had a median LOS of 18 days (81.6% prolonged hospitalisation), and those with pneumonia had a median LOS of 13 days (77.4%). Regarding procedures, mastectomy was associated with a median LOS of 6 days (27.6% prolonged hospitalisation), while inpatient radiotherapy (median 13 days; 70.3%) and complex intensive care (median 16.5 days; 87.5%) showed markedly longer stays (Table 2).

### 3.3. Primary Analysis: Characteristics Documented Independently of the In-Hospital Course and Hospital Length of Stay

This section reports the primary multivariable analysis, restricted to characteristics documented independently of the in-hospital course (Groups A and B, Section 2.2). In-hospital complications, procedures, and diagnoses of ambiguous timing are not included here; they are reported separately in Section 3.5 as secondary, descriptive associations.

**Figure 1:**
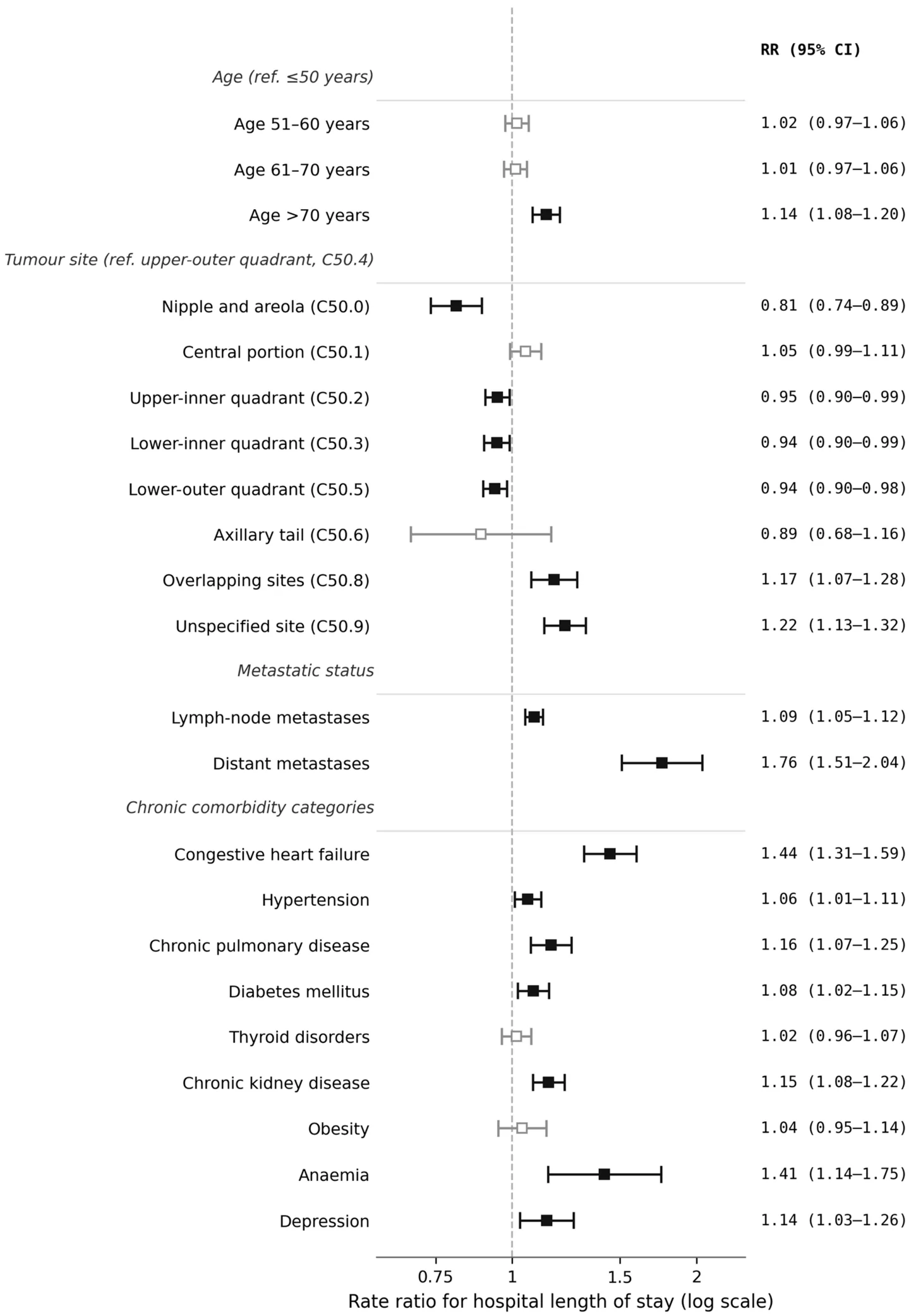
Primary analysis: association of characteristics documented independently of the in-hospital course with hospital length of stay in breast cancer hospitalisation episodes (multivariable negative binomial regression; rate ratios). Reference categories: age ≤ 50 years; tumour site upper-outer quadrant (C50.4). Cluster-robust (sandwich) standard errors, hospital as clustering variable (38 clusters).

In the multivariable negative binomial model (Figure 1), age > 70 years was associated with a longer LOS compared with age ≤ 50 years (RR 1.14; 95% CI 1.08–1.20). The age groups 51–60 years (RR 1.02; 95% CI 0.97–1.06) and 61–70 years (RR 1.01; 95% CI 0.97–1.06) were not significantly associated.

Tumour-site comparisons use the upper-outer quadrant (C50.4) as the reference category (see Section 2.3). Compared with this category, nipple/areola tumours (RR 0.81; 95% CI 0.74–0.89), upper-inner quadrant tumours (RR 0.95; 95% CI 0.90–0.99), lower-inner quadrant tumours (RR 0.94; 95% CI 0.90–0.99) and lower-outer quadrant tumours (RR 0.94; 95% CI 0.90–0.98) were associated with a shorter LOS, whereas overlapping sites (RR 1.17; 95% CI 1.07–1.28) and the unspecified site (RR 1.22; 95% CI 1.13–1.32) were associated with a longer LOS. Central portion and axillary tail tumours were not significantly associated.

Documented lymph node metastases (RR 1.09; 95% CI 1.05–1.12) and distant metastases (RR 1.76; 95% CI 1.51–2.04) were associated with a longer LOS.

Among the chronic comorbidity categories, congestive heart failure (RR 1.44; 95% CI 1.31–1.59), anaemia (RR 1.41; 95% CI 1.14–1.75), chronic pulmonary disease (RR 1.16; 95% CI 1.07–1.25), chronic kidney disease (RR 1.15; 95% CI 1.08–1.22), depression (RR 1.14; 95% CI 1.03–1.26), diabetes mellitus (RR 1.08; 95% CI 1.02–1.15) and hypertension (RR 1.06; 95% CI 1.01–1.11) were associated with a longer LOS. Thyroid disorders (RR 1.02; 95% CI 0.96–1.07) and obesity (RR 1.04; 95% CI 0.95–1.14) were not significantly associated.

Associations for in-hospital complications, procedures, and the two comorbidity categories of ambiguous timing are reported separately in Section 3.5.

The Poisson specification of the same model, reported in Table S2, yielded materially consistent estimates but showed clear evidence of overdispersion (see Section 2.3).

### 3.4. Primary Analysis: Characteristics Documented Independently of the In-Hospital Course and Prolonged Hospitalisation

As in Section 3.3, this section reports the primary analysis, restricted to characteristics documented independently of the in-hospital course; associations for in-hospital complications, procedures, and diagnoses of ambiguous timing are reported in Section 3.5. Tumour-site comparisons use the upper-outer quadrant (C50.4) as the reference category (see Section 2.3); using cluster-robust standard errors accounting for hospital clustering, the pattern and statistical significance of the associations reported below were essentially unchanged from the single-level estimates, and a full comparison, together with the negative binomial, alternative-cutoff, surgical-subgroup, admission-year, and comorbidity-exclusion sensitivity analyses, FDR-corrected *p*-values, and the hospital-exclusion sensitivity analysis, is reported in the Supplementary Materials.

**Figure 2:**
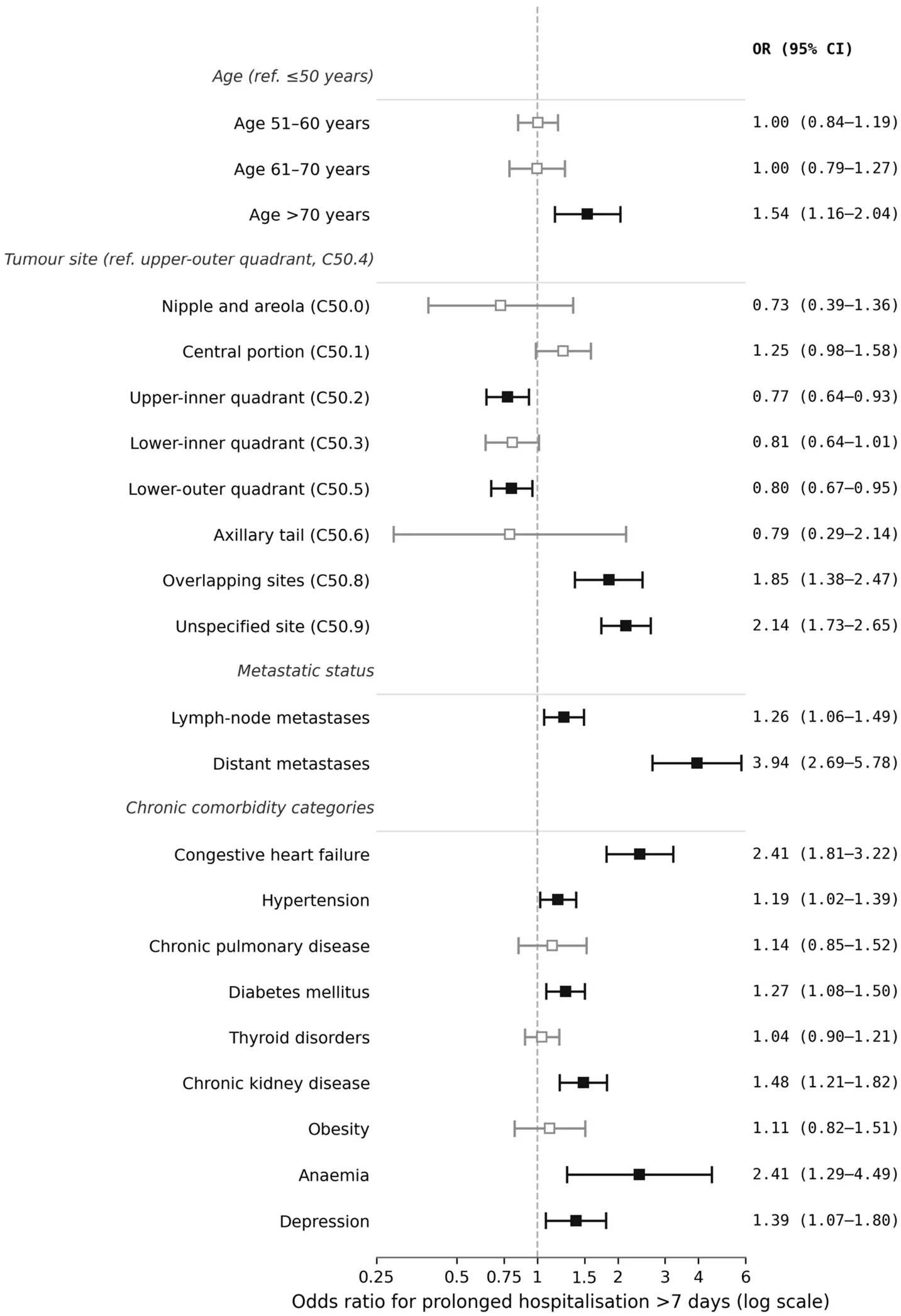
Primary analysis: association of characteristics documented independently of the in-hospital course with prolonged hospitalisation (>7 days) in breast cancer hospitalisation episodes (multivariable logistic regression; odds ratios). Reference categories: age ≤ 50 years; tumour site upper-outer quadrant (C50.4). Cluster-robust (sandwich) standard errors, hospital as clustering variable (38 clusters).

In the multivariable logistic model (Figure 2), age > 70 years was associated with prolonged hospitalisation compared with age ≤ 50 years (OR 1.54; 95% CI 1.16–2.04). The age groups 51–60 years (OR 1.00; 95% CI 0.85–1.19) and 61–70 years (OR 1.00; 95% CI 0.79–1.27) were not significantly associated.

Compared with the upper-outer quadrant, upper-inner quadrant tumours (OR 0.77; 95% CI 0.64–0.93) and lower-outer quadrant tumours (OR 0.80; 95% CI 0.67–0.96) were associated with a lower likelihood of prolonged hospitalisation, whereas overlapping sites (OR 1.85; 95% CI 1.38–2.47) and the unspecified site (OR 2.14; 95% CI 1.73–2.65) were associated with a higher likelihood. Nipple/areola, central portion, lower-inner quadrant and axillary tail tumours were not significantly associated.

Documented lymph node metastases (OR 1.26; 95% CI 1.06–1.49) and distant metastases (OR 3.94; 95% CI 2.69–5.78) were associated with prolonged hospitalisation.

Among the chronic comorbidity categories, congestive heart failure (OR 2.41; 95% CI 1.81–3.22), anaemia (OR 2.41; 95% CI 1.29–4.49), chronic kidney disease (OR 1.48; 95% CI 1.21–1.82), depression (OR 1.39; 95% CI 1.07–1.81), diabetes mellitus (OR 1.27; 95% CI 1.08–1.50) and hypertension (OR 1.19; 95% CI 1.02–1.39) were associated with prolonged hospitalisation. Chronic pulmonary disease (OR 1.14; 95% CI 0.85–1.52), thyroid disorders (OR 1.04; 95% CI 0.90–1.21) and obesity (OR 1.11; 95% CI 0.82–1.51) were not significantly associated.

The model discriminated moderately well (c = 0.750). Associations for in-hospital complications, procedures, and the two comorbidity categories of ambiguous timing are reported in Section 3.5.

Sensitivity analyses using ≥5-day and ≥7-day definitions of prolonged LOS, adjustment for admission year, and exclusion of the largest contributing hospital are reported in the Supplementary Materials; the direction and significance pattern of the associations above were consistent throughout, with the exception noted in Section 4.3.

### 3.5. Secondary Descriptive Analysis: In-Hospital Complications, Procedures, and Diagnoses of Ambiguous Timing

The analyses in this section add the in-hospital complications and procedures (Group D) and the two comorbidity categories whose timing cannot be established in these data (Group C) to the model specification of Section 3.3 and Section 3.4. Because these variables are documented during the hospitalisation whose length is the outcome, the estimates below describe the LOS profile of episodes in which these events were recorded. They are not predictors available at admission and must not be read as such.

**Figure 3:**
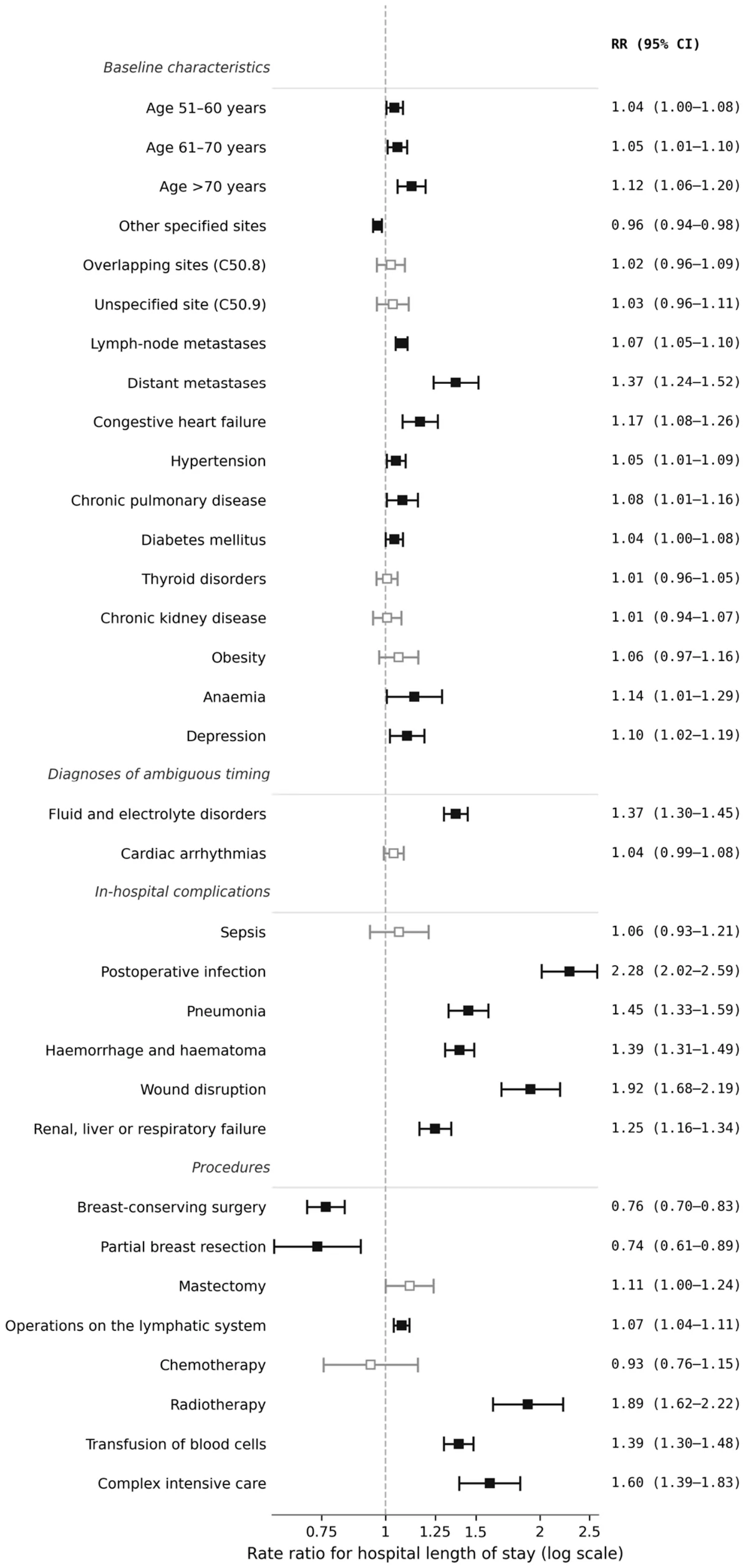
Secondary descriptive analysis: association of in-hospital complications, procedures, and diagnoses of ambiguous timing with hospital length of stay (multivariable negative binomial regression, adjusted for the characteristics of Figure 1; rate ratios). Cluster-robust (sandwich) standard errors, hospital as clustering variable (38 clusters). Tumour-site categories collapsed to three (see Section 3.5).

In the secondary model, the nine tumour-site categories were collapsed to three (other specified sites, overlapping sites, unspecified site; reference: upper-outer quadrant), so that the number of parameters remains below the number of hospital clusters (see Section 2.3). For hospital length of stay (Figure 3), the strongest associations were observed for postoperative infection (RR 2.28; 95% CI 2.02–2.59), wound disruption (RR 1.92; 95% CI 1.68–2.19), inpatient radiotherapy (RR 1.89; 95% CI 1.62–2.22), complex intensive care (RR 1.60; 95% CI 1.39–1.83), pneumonia (RR 1.45; 95% CI 1.33–1.58), transfusion of blood cells (RR 1.39; 95% CI 1.30–1.48), haemorrhage/haematoma (RR 1.39; 95% CI 1.31–1.49) and renal, liver or respiratory failure (RR 1.25; 95% CI 1.16–1.34). Fluid and electrolyte disorders were associated with a longer LOS (RR 1.37; 95% CI 1.30–1.45), whereas cardiac arrhythmias were not (RR 1.04; 95% CI 0.99–1.08). Breast-conserving surgery (RR 0.76; 95% CI 0.70–0.83) and partial breast resection (RR 0.74; 95% CI 0.61–0.89) were associated with a shorter LOS, mastectomy was not significantly associated (RR 1.11; 95% CI 1.00–1.24), and operations on the lymphatic system were associated with a slightly longer LOS (RR 1.07; 95% CI 1.04–1.11). Sepsis (RR 1.06; 95% CI 0.93–1.21) and chemotherapy (RR 0.93; 95% CI 0.76–1.15) were not significantly associated.

**Figure 4:**
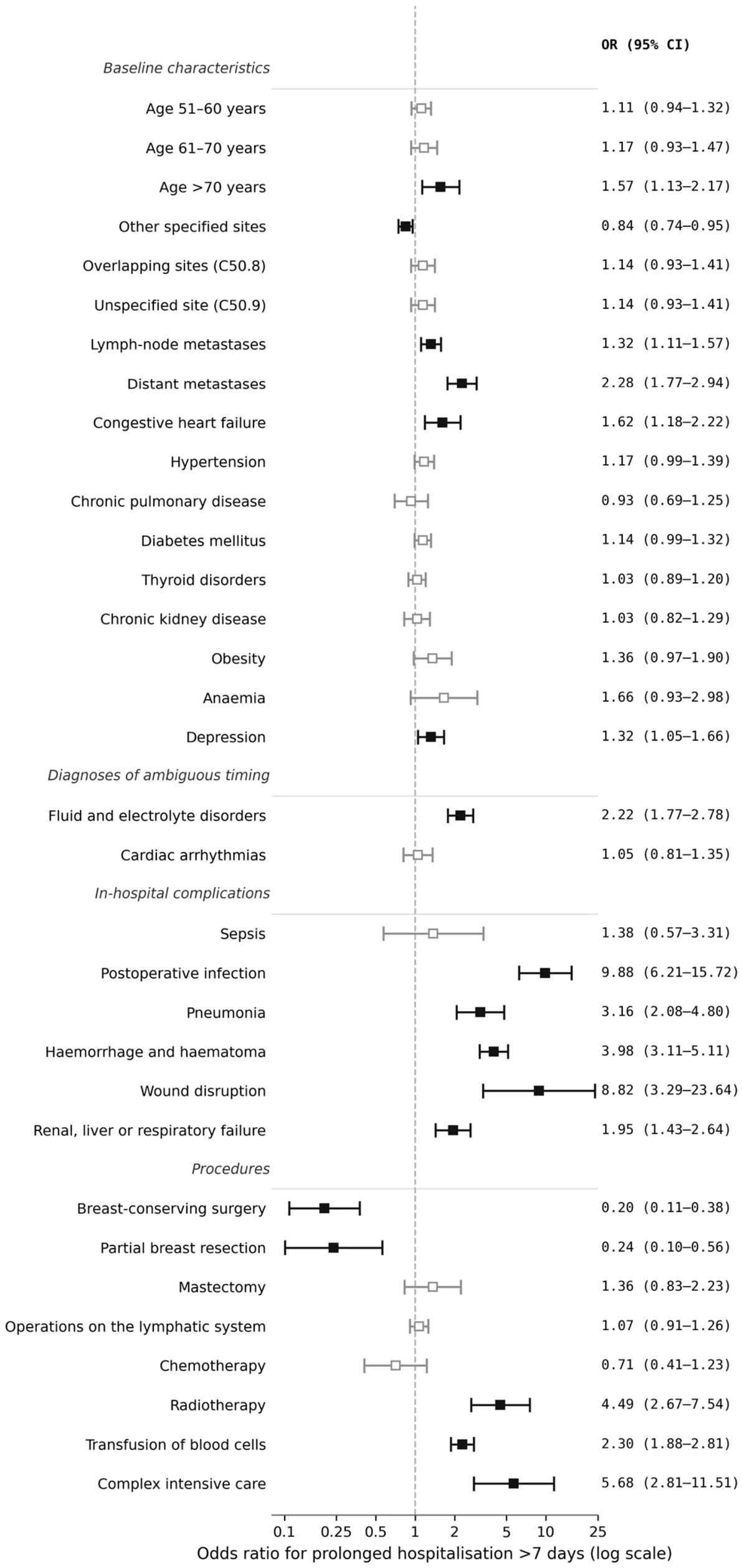
Secondary descriptive analysis: association of in-hospital complications, procedures, and diagnoses of ambiguous timing with prolonged hospitalisation (>7 days) (multivariable logistic regression, adjusted for the characteristics of Figure 2; odds ratios). Cluster-robust (sandwich) standard errors, hospital as clustering variable (38 clusters). Tumour-site categories collapsed to three (see Section 3.5).

For prolonged hospitalisation (Figure 4), the same pattern was more pronounced on the odds-ratio scale. Postoperative infection (OR 9.88; 95% CI 6.21–15.72), wound disruption (OR 8.82; 95% CI 3.29–23.64), complex intensive care (OR 5.68; 95% CI 2.81–11.51), inpatient radiotherapy (OR 4.49; 95% CI 2.67–7.54), haemorrhage/haematoma (OR 3.99; 95% CI 3.11–5.11), pneumonia (OR 3.16; 95% CI 2.08–4.80), transfusion of blood cells (OR 2.30; 95% CI 1.88–2.81) and renal, liver or respiratory failure (OR 1.95; 95% CI 1.43–2.64) were associated with a higher likelihood of a stay exceeding seven days. Fluid and electrolyte disorders were also associated with prolonged hospitalisation (OR 2.22; 95% CI 1.77–2.78), whereas cardiac arrhythmias were not (OR 1.05; 95% CI 0.81–1.36). Breast-conserving surgery (OR 0.20; 95% CI 0.11–0.38) and partial breast resection (OR 0.24; 95% CI 0.10–0.56) were associated with a lower likelihood, while mastectomy (OR 1.36; 95% CI 0.83–2.23), operations on the lymphatic system (OR 1.07; 95% CI 0.91–1.26), chemotherapy (OR 0.71; 95% CI 0.41–1.23) and sepsis (OR 1.38; 95% CI 0.57–3.32) were not significantly associated. The model discriminated well (c = 0.831). Re-estimating this model with the full nine-category tumour-site specification and a large-sample chi-square approximation gave virtually identical estimates for every covariate.

Two of these associations are close to circular by construction and should be read with that in mind. Complex intensive care is coded in the German OPS partly by the duration and intensity of care delivered. Inpatient radiotherapy accounts for only 1.9% of episodes; adjuvant radiotherapy after breast-conserving surgery is near-universal and almost entirely delivered on an outpatient basis in Germany, so this subgroup most likely represents predominantly palliative treatment for metastatic disease, or women too unwell for ambulatory care, rather than an association attributable to the treatment modality itself. The same reasoning applies to the small inpatient chemotherapy subgroup (2.1%), which probably reflects short scheduled infusion admissions. Sepsis was not significantly associated in either model, most likely reflecting the very low case count (*n* = 38; 0.2%) rather than an absence of association.

## 4. Discussion

### 4.1. Main Findings

This multicentre cross-sectional study demonstrates that longer hospital stays among women with breast cancer hospitalisation episodes are primarily associated with older age, metastatic disease and somatic comorbidities. Furthermore, acute complications and complex procedures were linked to a significantly longer length of stay, whereas breast-conserving surgery and partial resections were clearly associated with shorter stays. The former (age, metastatic status, chronic comorbidities) are characteristics documented independently of the in-hospital course and constitute the primary analysis; the latter (complications, intensive procedures, and diagnoses of ambiguous timing) are documented during the hospitalisation and are reported as secondary descriptive correlates rather than predictors (see Section 4.3).

### 4.2. Comparison with Previous Studies

In this multicentre German study, the median length of hospital stay for breast cancer patients was 4 days (IQR 3–7 days). Our findings are therefore consistent with the pattern described by Marla et al. [8] in Glasgow. In that study, the median length of stay was 1 day for screen-detected tumours and 4 days for symptomatic tumours. Depending on the procedure performed, the hospital stay was 1 day following breast-conserving surgery, 5 days following a mastectomy, and 7 days following a mastectomy with reconstruction. Downing et al. [7] reported, based on a large population-based study from two English regions, a median hospital stay of 3 days following breast-conserving surgery and 5 days following a mastectomy. The study noted a reduction in the length of stay (LOS) between 1997/1998 and 2004/2005, totalling one day; however, this was attributed more to organisational changes, such as an increase in admissions on the day of surgery and altered discharge criteria, than to the shift from mastectomy to breast-conserving surgery, which accounted for only about 9% of the reduction [7]. In the analysis by Gümüş et al. [12], the average postoperative LOS following breast cancer surgery was found to be more than 6 days. This longer LOS is associated in particular with extensive axillary procedures [12]. Fundamentally, these studies demonstrate that the LOS observed in the present study is consistent with the findings of other European studies, not only in terms of absolute duration but also with regard to the differences between breast-conserving surgery and mastectomy.

It should be noted that the study by Gümüş et al. [12] was a single-centre study from Turkey, reflecting a different healthcare system and patient population from the present German multicentre analysis; comparisons with that study should be made with appropriate caution. The analyses by Gümüş et al. [12], Marla et al. [8] and Downing et al. [7] consistently indicate that tumour stage, the extent of axillary procedures and the type of treatment are factors consistently associated with the length of hospital stay, a finding that is also confirmed by the present study.

The findings of this study are largely consistent with those of the study by Gümüş et al. [12], who demonstrated in a Turkish single-centre study that the presence and number of metastases in the lymph nodes and the duration of drainage placement are important independent variables associated with a longer length of hospital stay, whereas age did not appear to be significantly associated [12]. Although the present study did not directly record the duration of drainage placement, there is nevertheless a significant association between a longer LOS and operations on the lymphatic system, which in clinical practice usually involve the insertion of an axillary drain. Thus, the associations highlighted by Gümüş et al. [12] between axillary tumour burden and procedures requiring drainage and a prolonged hospital stay can be indirectly substantiated, even though drainage was not recorded as a separate parameter in the study.

Studies by Downing et al. [7] and Marla et al. [8] from the UK further illustrate that mastectomies, axillary procedures, older age, advanced cancer stage and comorbidities are associated with longer hospital stays, whereas breast-conserving treatments are associated with shorter hospital stays. Similar findings are also evident in the present study. Here, it was established that breast-conserving procedures and partial resections are linked to significantly shorter stays and a clearly reduced likelihood of prolonged hospitalisation. In contrast, mastectomy, lymph node surgery, blood transfusions and intensive care treatments were associated with a longer LOS. In contrast to the analysis by Marla et al. [8], which is based on data from five hospitals in Glasgow, and the population-based study by Downing et al. [7], whose data originate from two English regions, this analysis is based on a large, Germany-wide and up-to-date database and thus covers a wide range of hospitals and treatment settings.

Furthermore, these findings can be contextualised alongside additional international analyses of complex procedures, comorbidities and complications. Kotha et al. [13] and Jonczyk et al. [14] show that complex reconstructive procedures, longer operating times, the need for transfusions and postoperative complications are particularly associated with a prolonged LOS. Dehal et al. [15], Park et al. [16] and Hall et al. [17] further highlight the significance of serious complications and sepsis, which can be associated with a significantly prolonged length of stay and increased mortality.

Kotha et al. [13] demonstrated, using a national dataset from the USA, that longer operating times, obesity, diabetes mellitus, transfusions and early postoperative complications are significant factors associated with a prolonged hospital stay. Similar process-oriented associations can be derived from the data analysed here. Comorbidities such as obesity and diabetes mellitus correlated with a longer LOS. Transfusions and acute complications were strongly associated with a longer LOS and with a higher likelihood of a hospital stay exceeding 7 days. Whilst Kotha et al. [13] investigated autologous breast reconstruction and, as a result, examined a highly selective cohort, the present study suggests that comparable associations can also be observed in a broader cohort of breast cancer patients who were treated as inpatients in different hospitals.

In their analysis of women with breast cancer treated in hospitals, Park et al. [16] report that a concurrent diagnosis of heart failure is associated with increased in-hospital mortality, longer hospital stays and higher costs. Another study by Dehal et al. [15] is based on the modified Charlson Comorbidity Index according to Deyo and demonstrated significant correlations between the degree of comorbidity, postoperative complications and prolonged hospital stays in women following breast cancer surgery. In the modified Charlson score according to Deyo, 17 predefined comorbidities are combined with a corresponding weighting factor to form a total score that defines the overall degree of morbidity in patients [15].

Unlike in the study by Dehal et al. [15], comorbidities in the present study were recorded using individual comorbidity categories defined by the Elixhauser classification rather than a summary index score. This classification defines 31 individual comorbidity categories, covering a broad spectrum of somatic and mental comorbidities, thereby allowing a more nuanced analysis of the relationships between specific pre-existing conditions and LOS [18]. On this basis, it was demonstrated that, amongst others, heart failure and renal failure, diabetes mellitus, obesity, hypertension, electrolyte disturbances and depression were associated with a significantly longer hospital stay and a higher probability of an extended LOS. The present analysis can thus complement studies based on the Charlson Comorbidity Index by demonstrating that, through a differentiated assessment of comorbidities using the Elixhauser approach, clear correlations can be established between comorbidities and the duration of inpatient treatment and, consequently, resource consumption among breast cancer patients in Germany.

With regard to complications, there is also clear consistency with the international evidence. A large-scale registry data analysis by Jonczyk et al. [14] demonstrates that transfusions, infections, wound healing disorders and reoperations constitute the most significant acute complications following breast cancer surgery and are thus closely associated with the postoperative course, being accompanied by additional interventions and prolonged recovery times. The present study showed that infections, wound healing disorders, haematomas or bleeding, pneumonia and organ failure showed the strongest associations with LOS and with the likelihood of a hospital stay exceeding 7 days. In the multivariate models, sepsis showed no statistical significance in relation to LOS. This may be due to the very low number of cases, at just 0.2%, which must be taken into account as a possible reason for the lack of evidence of an association. At the same time, the descriptive results showing a median length of stay of 18 days and a high proportion of prolonged hospital stays (81.6%) suggest that sepsis has considerable clinical relevance in the inpatient course of affected patients.

The large-scale national analysis by Hall et al. [17] demonstrates that sepsis is generally associated with a longer LOS. This US study shows that the LOS for patients with sepsis as the primary diagnosis is up to 75% longer than for other inpatients. From this perspective, it seems plausible that sepsis may also be associated with prolonged courses in the cohort examined here, and that the lack of statistical significance is primarily attributable to the small sample size rather than the absence of an actual association.

### 4.3. Strengths and Limitations

A key strength of this study is its extensive, up-to-date dataset, which covered 16,062 breast cancer hospitalisation episodes between 2019 and 2024 in 38 hospitals offering different levels of care. Treatment approaches ranging from university-based maximum care through specialist and standard care to care in specialised facilities were examined. This ensured a high degree of heterogeneity in the case-mix and care structures analysed.

In addition, routine hospital data was used, standardised and transferred in a uniform format. This ensured a reliable representation of diagnoses, procedures and LOS across a large number of hospitals. It also helped to reduce selection-related biases arising from case selection, which can occur in voluntary or single-centre studies.

A further strength of the analysis is the detailed recording of comorbidities using the individual comorbidity categories defined by the Elixhauser classification. This classification comprises 31 defined categories of comorbidities, thereby enabling the detailed recording of individual pre-existing conditions. Unlike global summary scores, this approach enables the identification of specific comorbidity profiles associated with prolonged hospital stays and the risk of extended LOS. This is complemented by the use of multivariable negative binomial and logistic regression models, which enable a robust assessment of independent associations, as they account for potential confounders such as age, tumour location and comorbidities.

In addition to its strengths, the study also has a number of limitations that must be taken into account when interpreting the results. As this study is based on routinely collected billing data, the information is presented in the form of ICD and OPS codes. Important clinical details, such as tumour biology in the form of receptor status and grading, functional status or home care, are therefore not considered. Discharge targets or post-hospital care arrangements could not be mapped or included either, although these may influence the length of hospital stay [19]. Furthermore, no information was collected on socio-economic characteristics such as educational attainment, employment status or type of health insurance, whereas a study by Yong and Yang did find a relevant association with LOS in this regard [20].

Furthermore, routine data such as that used in this study may be subject to information bias, as it is prone to coding errors, under-reporting or institutional differences in coding practices. Complications are particularly noteworthy in this regard, as they may not necessarily be interpreted as treatment-related complications but rather as secondary diagnoses. Furthermore, comorbidities of low severity may be underrepresented in the billing context. Furthermore, only comorbidity categories documented in at least 1% of the study population were included in the Elixhauser analysis. While chosen for analytical stability, this threshold may have led to underrepresentation of rare but clinically relevant conditions that could independently influence hospital length of stay. In addition, present-on-admission information was not available, so it is not possible to determine with certainty whether individual comorbidity codes were recorded before or during the hospitalisation. This is a fundamental constraint of §21 data and cannot be resolved analytically: for some categories the timing is clinically unambiguous, for others it can only be assumed. We have therefore avoided the claim that any diagnosis was “known at admission”, and instead restricted the primary model to categories that are chronic by definition, while the two categories most plausibly subject to de novo onset during an inpatient stay (fluid/electrolyte disorders, cardiac arrhythmias) were moved to the secondary analysis; a sensitivity analysis re-including them is reported in the Supplementary Materials. Residual misclassification of timing within the retained chronic categories cannot be excluded. We also note that several covariates in the multivariable models (in particular in-hospital complications and procedures) are not conceptualised as confounders of any single exposure–LOS relationship, so individual coefficients should not be interpreted as isolated causal effect estimates (the “Table 2 fallacy”).

Clustering of hospitalisation episodes within hospitals was addressed using cluster-robust standard errors (cross-validated against multilevel models), given the considerable heterogeneity in case volume across the 38 participating hospitals (median 110 cases, IQR 7–655); one hospital, likely a tertiary/university centre, alone contributed 23.4% of admissions, and a sensitivity analysis excluding this hospital showed that most associations were stable, with the exception of breast-conserving surgery, whose association with shorter LOS was materially attenuated when this hospital was excluded, consistent with a centre-specific fast-track practice at this site. A residual limitation is that, because patient-level linkage was not possible (see Section 2), any non-independence between repeat admissions by the same woman could not be addressed, only between-hospital clustering. Given the cross-sectional and retrospective observational study design used in this analysis, it is not possible to draw causal conclusions in the form of a cause-and-effect relationship, but rather to describe statistical associations. This limits the study, as does the sometimes very low number of cases for certain rare events. Sepsis, for example, occurred so rarely that the statistical power to identify independent effects is significantly limited. Against this background, the lack of statistical significance for rare complications should not be interpreted as an indication that there is no clinically relevant association with the length of hospital stay. This applies in particular to in-hospital complications and procedures, which are not baseline characteristics but events documented during the hospitalisation whose length is the outcome, and which may lie on the pathway between admission and LOS or represent a consequence of a longer stay rather than an antecedent of it. For this reason, they are reported separately, as secondary descriptive associations. A further, more fundamental constraint is that only 38 hospitals contributed data. In the secondary model this is binding rather than merely inconvenient: with the full nine-category tumour-site specification, the number of parameters (40) exceeds the number of clusters (38), so the design-based tests for the cluster-robust variance estimator cannot be computed. The tumour-site categories were therefore collapsed to three in the secondary model only, keeping the number of parameters below the number of clusters; the primary model retains the full resolution. Even so, this leaves very little residual degrees of freedom for the cluster-robust variance estimator; this is a known limitation of cluster-robust and related clustering-correction methods when the number of clusters is small relative to the number of covariates, and it applies to essentially any clustering-correction method (GEE, multilevel, or cluster-robust) given this number of hospitals. The primary model reported in Figure 1 and Figure 2 is considerably more parsimonious and therefore has a more favourable ratio of hospitals to parameters; the cluster-robust estimates of the secondary model should nonetheless be interpreted with this constraint in mind.

### 4.4. Interpretation and Clinical Implications

The findings of this study show that differences in the length of hospital stay among breast cancer hospitalisation episodes are associated both with characteristics documented independently of the in-hospital course and with events documented during the hospitalisation itself. Older age, metastatic disease, and certain chronic comorbidities are associated with prolonged hospital stays and are, in most instances, plausibly present before admission; acute complications and complex procedures show the strongest associations of all, but as in-hospital events, they cannot themselves be used to identify, at admission, which episodes will go on to be prolonged.

For everyday clinical practice, the baseline factors identified here (age, metastatic disease, comorbidity burden) may help identify hospitalisation episodes at elevated risk of a prolonged stay at the time of admission. Structured perioperative management, proactive management of comorbidities, and optimised discharge planning may accordingly be of value for these episodes; because the largest estimates in this study belong to in-hospital complications and intensive procedures that are documented only during the stay, risk-adapted pathways should be built around the process-of-care factors available at or before admission rather than around the complications themselves. In addition to a potentially more efficient use of inpatient resources, such measures may also support patient safety.

There are also promising avenues of research that could address not only the length of hospital stay but also clinical outcomes. This could, for example, take the form of developing a predictive model to estimate the duration of hospitalisation. The development of specific care programmes, such as multimodal treatment approaches for patients with a high burden of comorbidity, could also be considered.

## 5. Conclusions

In this large, contemporary, multicentre German cohort of 16,062 breast cancer hospitalisation episodes, the primary analysis of characteristics documented independently of the in-hospital course showed that prolonged hospital stay was associated with age > 70 years, distant and lymph node metastases, and chronic comorbidities including congestive heart failure, anaemia, chronic kidney disease, depression, diabetes mellitus and hypertension. Thyroid disorders and obesity were not associated with LOS in either primary model. In secondary descriptive analyses, the strongest associations of all were observed for in-hospital complications (particularly postoperative infection, wound disruption and pneumonia), inpatient radiotherapy, and intensive procedures (intensive care, transfusion); fluid and electrolyte disorders, whose timing cannot be established in these data, also fall into this secondary group. Breast-conserving surgery and partial breast resection were consistently associated with shorter stays, including within the subgroup of surgical admissions. These associations were robust to cluster-robust adjustment for hospital-level clustering, alternative definitions of prolonged LOS, and correction for multiple testing, although a small number of comorbidity associations (hypertension, obesity, diabetes) did not survive these robustness checks. Because the strongest correlates of prolonged stay are events documented during the hospitalisation rather than characteristics documented independently of it, and because this cross-sectional, retrospective design does not support causal inference, these findings should be read as identifying clinical and procedural correlates of resource use—useful for perioperative planning and discharge management—rather than as a basis for predicting, at admission, which individual women will experience a prolonged stay.

## Figures and Tables

**Table 1:** Baseline characteristics of the study sample (hospitalisation episodes).

Variable	N (%) or Mean (SD) (*n* = 16,062)
Age	
Mean age (SD)	62.8 (14.0)
≤50 years, *n* (%)	3386 (21.1)
51–60 years, *n* (%)	3690 (23.0)
61–70 years, *n* (%)	3876 (24.1)
>70 years, *n* (%)	5110 (31.8)
Site of breast cancer, *n* (%)	
Nipple and areola	119 (0.7)
Central portion	959 (6.0)
Upper-inner quadrant	1597 (9.9)
Lower-inner quadrant	929 (5.8)
Upper-outer quadrant	5863 (36.5)
Lower-outer quadrant	1371 (8.5)
Axillary tail of breast	31 (0.2)
Overlapping sites	3098 (19.3)
Unspecified site	2095 (13.0)
Lymph node metastases (C77), *n* (%)	3497 (21.8)
Distant metastases, *n* (%)	2758 (17.2)
Comorbidities, *n* (%)	
Congestive heart failure	349 (2.2)
Cardiac arrhythmias	883 (5.5)
Hypertension	5921 (36.9)
Chronic pulmonary disease	319 (2.0)
Diabetes mellitus	1462 (9.1)
Thyroid gland disorders	2627 (16.4)
Chronic kidney disease	471 (2.9)
Obesity	840 (5.2)
Anaemia	187 (1.2)
Fluid and electrolyte disorders	1255 (7.8)
Depression	509 (3.2)
Complications, *n* (%)	
Sepsis	38 (0.2)
Infections	68 (0.4)
Haemorrhage and haematoma	463 (2.9)
Disruption of wound	39 (0.2)
Pneumonia	234 (1.5)
Renal, liver or respiratory failure	666 (4.2)
Procedures, *n* (%)	
Breast-conserving surgery	2214 (13.8)
Partial breast resection	5664 (35.3)
Mastectomy	3425 (21.3)
Operations on the lymphatic system	9991 (62.2)
Chemotherapy	330 (2.1)
Radiotherapy	300 (1.9)
Transfusion of blood cells	642 (4.0)
Complex intensive care	112 (0.7)

**Table 2:** Median hospital length of stay (days) and proportion of hospitalisation episodes with prolonged hospitalisation among women with breast cancer.

Patient Group	Median (IQR)	>7 Days (%)
**Total**	**4 (3–7)**	**19.3**
Age		
≤50 years	4 (3–6)	14.4
51–60 years	4 (3–6)	15.9
61–70 years	4 (3–6)	17.0
>70 years	5 (3–8)	26.9
Site of breast cancer		
Nipple and areola	3 (2–5)	13.5
Central portion	5 (3–7)	19.5
Upper-inner quadrant	4 (3–5)	11.2
Lower-inner quadrant	4 (3–6)	12.2
Upper-outer quadrant	4 (3–6)	14.0
Lower-outer quadrant	4 (3–6)	12.0
Axillary tail of breast	4 (3–7)	16.1
Overlapping sites	5 (3–8)	28.5
Unspecified site	6 (3–9)	35.1
Lymph node metastases (C77)	5 (3–7)	25.0
Distant metastases	7 (4–12)	47.3
Comorbidities		
Congestive heart failure	8 (4–15)	53.6
Cardiac arrhythmias	5 (3–9)	33.3
Hypertension	5 (3–7)	24.1
Chronic pulmonary disease	6 (4–10)	32.6
Diabetes mellitus	5 (3–8)	30.7
Thyroid gland disorders	4 (3–7)	21.6
Chronic kidney disease	7 (4–13)	48.0
Obesity	4 (3–7)	22.3
Anaemia	7 (4–11)	43.9
Fluid and electrolyte disorders	9 (5–15)	58.3
Depression	5 (3–8)	29.1
Complications		
Sepsis	17.5 (10–29)	81.6
Infections	13 (6–24)	70.6
Haemorrhage and haematoma	7 (5–9)	39.1
Disruption of wound	15 (8–22)	76.9
Pneumonia	13 (8–21)	77.4
Renal, liver or respiratory failure	11 (6–17)	65.9
Procedures		
Breast-conserving surgery	4 (3–5)	4.7
Partial breast resection	3 (2–5)	5.2
Mastectomy	6 (4–8)	27.6
Operations on the lymphatic system	4 (3–6)	11.8
Chemotherapy	4 (2–12)	36.7
Radiotherapy	13 (6.5–21.5)	70.3
Transfusion of blood cells	9 (6–17)	61.5
Complex intensive care	16.5 (9–27.5)	87.5

## Data Availability

The data presented in this study are available on request from the corresponding author. The data are not publicly available due to privacy restrictions.

## Supplementary Materials

The following supporting information can be downloaded at: https://www.mdpi.com/article/10.3390/clinpract16090169/s1, Table S1: Overdispersion diagnostics; Table S2: Poisson sensitivity analysis of the primary length-of-stay model; Table S3: Alternative definitions of prolonged length of stay; Table S4: Surgical admissions; Table S5: Admission-year adjustment; Table S6: Sensitivity analysis adding the comorbidity categories of ambiguous timing; Table S7: Sensitivity analysis excluding the largest hospital; Table S8: Correction for multiple testing; Table S9: STROBE Statement.
