# Supplementary material for: Factors Associated with Prolonged Hospitalisation in Women with Breast Cancer: Evidence from a Large German Multicentre Study
Source: Clin Pract. 2026 Sep 11;16(9):169. doi: 10.3390/clinpract16090169 (PMC13605783; doi:10.3390/clinpract16090169)
Supplement: Supplementary file 1 [file clinpract-16-00169-s001.zip › clinpract-4508447-supplementary.pdf]

Supplementary Material

Factors Associated with Prolonged Hospitalisation in Women with Breast Cancer: Evidence from a Large German Multicentre Study

All estimates in this Supplementary Material are taken from the same final set of models as the main text and its figures. Unless stated otherwise, models use cluster-robust (sandwich) standard errors with an independence working correlation and hospital as the clustering variable (38 clusters). The primary model comprises age, tumour site, lymph-node and distant metastases, and the chronic comorbidity categories; the secondary model additionally includes the two comorbidity categories of ambiguous timing and the in-hospital complications and procedures.

Table S1. Overdispersion diagnostics

Goodness-of-fit statistics for the Poisson and negative binomial specifications of the length-of-stay model, fitted without clustering so that likelihood-based fit criteria are comparable. The negative binomial model was adopted as the primary length-of-stay model on this basis; the Poisson model is reported as a sensitivity analysis in Table S2.

Model	Deviance/df	Pearson χ^2/df	AIC
Poisson	1.742	2.081	82,481.2
Negative binomial	0.855	1.064	76,695.3

Values close to 1 indicate an adequate mean-variance relationship; values well above 1 indicate overdispersion.

Table S2. Poisson sensitivity analysis of the primary length-of-stay model

The primary model of Figure 1 re-estimated with a Poisson rather than a negative binomial distribution, with the same covariates and the same clustering. Rate ratios were materially unchanged; the confidence intervals are narrower, consistent with the overdispersion documented in Table S1.

Variable	RR (95% CI), negative binomial	RR (95% CI), Poisson
Age 51-60 years	1.02 (0.97–1.06)	1.02 (0.97–1.07)
Age 61-70 years	1.01 (0.97–1.06)	1.01 (0.98–1.05)
Age >70 years	1.14 (1.08–1.20)	1.13 (1.07–1.18)
Nipple and areola (C50.0)	0.81 (0.74–0.89)	0.80 (0.72–0.90)
Central portion (C50.1)	1.05 (0.99–1.11)	1.04 (0.98–1.10)
Upper-inner quadrant (C50.2)	0.95 (0.90–0.99)	0.95 (0.90–1.00)
Lower-inner quadrant (C50.3)	0.94 (0.90–0.99)	0.95 (0.90–1.00)
Lower-outer quadrant (C50.5)	0.94 (0.90–0.98)	0.94 (0.89–0.98)
Axillary tail (C50.6)	0.89 (0.68–1.16)	0.87 (0.68–1.13)
Overlapping sites (C50.8)	1.17 (1.07–1.28)	1.17 (1.07–1.27)
Unspecified site (C50.9)	1.22 (1.13–1.32)	1.19 (1.10–1.30)
Distant metastases	1.76 (1.51–2.04)	1.75 (1.51–2.04)
Lymph-node metastases	1.09 (1.05–1.12)	1.08 (1.05–1.12)
Congestive heart failure	1.44 (1.31–1.60)	1.40 (1.26–1.54)
Hypertension	1.06 (1.01–1.11)	1.06 (1.01–1.12)
Chronic pulmonary disease	1.16 (1.07–1.25)	1.14 (1.05–1.23)
Diabetes mellitus	1.08 (1.02–1.15)	1.07 (1.00–1.15)
Thyroid disorders	1.02 (0.96–1.07)	1.02 (0.96–1.08)
Chronic kidney disease	1.15 (1.08–1.22)	1.14 (1.07–1.21)
Obesity	1.04 (0.95–1.14)	1.03 (0.96–1.11)
Anaemia	1.41 (1.14–1.75)	1.45 (1.19–1.76)
Depression	1.14 (1.03–1.26)	1.14 (1.04–1.27)

Reference categories: age ≤50 years; tumour site upper-outer quadrant (C50.4).

Table S3. Alternative definitions of prolonged length of stay

The primary logistic model repeated using ≥5-day and ≥7-day definitions of prolonged length of stay in addition to the primary >7-day (75th percentile) definition. All covariates are reported; no cells are omitted.

Variable	OR (≥5 days)	OR (≥7 days)	OR (>7 days, primary)
Age 51-60 years	1.04 (0.93–1.18)	0.95 (0.79–1.14)	1.00 (0.85–1.19)
Age 61-70 years	1.07 (0.88–1.29)	0.97 (0.78–1.19)	1.00 (0.79–1.26)
Age >70 years	1.65 (1.36–1.99)	1.53 (1.24–1.90)	1.54 (1.17–2.02)
Nipple and areola (C50.0)	0.63 (0.42–0.94)	0.58 (0.36–0.95)	0.73 (0.40–1.33)
Central portion (C50.1)	1.26 (1.02–1.55)	1.24 (1.00–1.55)	1.25 (0.99–1.57)
Upper-inner quadrant (C50.2)	0.80 (0.65–1.00)	0.78 (0.65–0.95)	0.77 (0.65–0.92)
Lower-inner quadrant (C50.3)	0.79 (0.65–0.96)	0.81 (0.61–1.08)	0.81 (0.65–1.01)
Lower-outer quadrant (C50.5)	0.84 (0.70–1.01)	0.78 (0.61–1.01)	0.80 (0.67–0.95)
Axillary tail (C50.6)	0.94 (0.29–3.08)	1.12 (0.45–2.76)	0.79 (0.30–2.07)
Overlapping sites (C50.8)	1.32 (0.99–1.74)	1.54 (1.19–2.00)	1.85 (1.39–2.45)
Unspecified site (C50.9)	1.68 (1.28–2.21)	2.09 (1.66–2.63)	2.14 (1.74–2.64)
Distant metastases	2.49 (1.74–3.57)	3.45 (2.38–4.98)	3.94 (2.72–5.71)
Lymph-node metastases	1.43 (1.19–1.71)	1.32 (1.15–1.51)	1.26 (1.06–1.48)
Congestive heart failure	1.85 (1.39–2.45)	2.20 (1.74–2.78)	2.41 (1.83–3.19)
Hypertension	1.18 (1.01–1.38)	1.17 (1.00–1.38)	1.19 (1.03–1.39)
Chronic pulmonary disease	1.27 (0.89–1.82)	1.18 (0.93–1.49)	1.14 (0.86–1.51)
Diabetes mellitus	1.13 (0.98–1.29)	1.29 (1.09–1.52)	1.27 (1.09–1.50)
Thyroid disorders	0.90 (0.79–1.04)	1.01 (0.88–1.16)	1.04 (0.90–1.20)
Chronic kidney disease	1.35 (1.12–1.63)	1.42 (1.19–1.69)	1.48 (1.22–1.81)
Obesity	1.06 (0.67–1.68)	1.11 (0.80–1.53)	1.11 (0.83–1.49)
Anaemia	1.95 (1.23–3.10)	2.39 (1.40–4.10)	2.41 (1.32–4.40)
Depression	1.41 (1.01–1.96)	1.42 (1.05–1.91)	1.39 (1.08–1.79)

Direction and statistical significance were consistent across all three definitions.

Table S4. Surgical admissions

30.6% of episodes (4,920/16,062) had no coded breast-surgery procedure. The model was repeated restricted to the 11,142 (69.4%) episodes with a coded surgical procedure. Non-surgical episodes had a median length of stay of 6 days, surgical episodes 4 days. Because only 26 of the 38 hospitals contributed surgical episodes, a more parsimonious specification was used, keeping the number of hospitals above the number of parameters. Mastectomy is the reference category for surgical category, since no all-category-zero reference group exists within this subgroup. Cluster-robust standard errors, 26 clusters.

Variable	OR (95% CI)	p
Age 51-60 years	1.24 (0.95–1.62)	0.1115
Age 61-70 years	1.30 (0.88–1.93)	0.1880
Age >70 years	2.23 (1.30–3.83)	0.0037
Distant metastases	1.45 (1.07–1.96)	0.0156
Lymph-node metastases	1.47 (1.18–1.84)	0.0007
Breast-conserving surgery	0.15 (0.07–0.31)	<0.0001
Partial breast resection	0.17 (0.09–0.34)	<0.0001
Operations on the lymphatic system	1.57 (1.33–1.84)	<0.0001
Postoperative infection	30.11 (12.49–72.61)	<0.0001
Pneumonia	15.85 (4.05–62.05)	<0.0001

Haemorrhage and haematoma	4.34 (3.40–5.54)	<0.0001
Renal, liver or respiratory failure	2.70 (1.75–4.16)	<0.0001
Transfusion of blood cells	4.36 (2.53–7.52)	<0.0001
Complex intensive care	19.36 (5.71–65.67)	<0.0001

Sepsis and wound disruption were excluded from this model (too few events for stable estimation within this subgroup). Complication estimates are numerically larger than in the full-cohort model, plausibly because the shorter baseline length of stay typical of surgical episodes makes a complication a proportionally larger deviation.

Table S5. Admission-year adjustment

The primary logistic model additionally adjusted for admission year (reference: 2024), to account for potential COVID-19-related changes in admission and discharge practice. The admission-year variable records the year of admission; four transfer episodes were admitted before 1 January 2019 and discharged within the study period, which is why the variable ranges from 2018 to 2024 while the study period is 2019–2024. Associations for the remaining covariates were materially unchanged.

**Admission year**	**OR (95% CI), ref. 2024**	**p**
2018 (n=4)	13.84 (1.44–132.84)	0.0228
2019	1.53 (1.21–1.94)	0.0004
2020	1.46 (0.99–2.15)	0.0592
2021	1.43 (1.11–1.85)	0.0053
2022	1.40 (1.09–1.82)	0.0095
2023	1.14 (0.90–1.44)	0.2694

A general trend toward shorter admissions across the study period is apparent; this trend spans, but is not specific to, the COVID-19 pandemic years. The 2018 estimate rests on four episodes and should not be interpreted.

Table S6. Sensitivity analysis adding the comorbidity categories of ambiguous timing

Fluid and electrolyte disorders and cardiac arrhythmias cannot be assigned to a time before or during the hospitalisation in these data and are therefore excluded from the primary model by design. This analysis re-estimates the primary model with both categories added, to show that their omission does not materially alter the associations for the remaining, chronic-by-definition comorbidities.

**Variable**	**OR (95% CI), primary model**	**OR (95% CI), with both categories added**
Congestive heart failure	2.41 (1.83–3.19)	2.07 (1.47–2.92)
Hypertension	1.19 (1.03–1.39)	1.16 (1.00–1.34)
Chronic pulmonary disease	1.14 (0.86–1.51)	1.09 (0.84–1.41)
Diabetes mellitus	1.27 (1.09–1.50)	1.23 (1.06–1.42)
Thyroid disorders	1.04 (0.90–1.20)	1.02 (0.88–1.17)
Chronic kidney disease	1.48 (1.22–1.81)	1.18 (0.97–1.44)
Obesity	1.11 (0.83–1.49)	1.16 (0.87–1.55)
Anaemia	2.41 (1.32–4.40)	2.21 (1.20–4.07)
Depression	1.39 (1.08–1.79)	1.32 (1.03–1.69)
Fluid and electrolyte disorders	– (not in primary model)	3.52 (2.89–4.29)
Cardiac arrhythmias	– (not in primary model)	1.15 (0.87–1.53)

Table S7. Sensitivity analysis excluding the largest hospital

One hospital contributed 3,764/16,062 episodes (23.4%). The primary logistic model was repeated excluding this hospital (n=12,298; 37 clusters) to assess whether the associations were attributable to this single, disproportionately large centre.

Variable	OR (95% CI), full cohort	OR (95% CI), excluding largest hospital
Age 51-60 years	1.00 (0.85–1.19)	1.08 (0.92–1.27)
Age 61-70 years	1.00 (0.79–1.26)	1.13 (0.95–1.34)
Age >70 years	1.54 (1.17–2.02)	1.78 (1.42–2.21)
Nipple and areola (C50.0)	0.73 (0.40–1.33)	0.78 (0.38–1.60)
Central portion (C50.1)	1.25 (0.99–1.57)	1.19 (0.84–1.69)
Upper-inner quadrant (C50.2)	0.77 (0.65–0.92)	0.81 (0.67–0.98)
Lower-inner quadrant (C50.3)	0.81 (0.65–1.01)	0.87 (0.68–1.12)
Lower-outer quadrant (C50.5)	0.80 (0.67–0.95)	0.82 (0.68–0.99)
Axillary tail (C50.6)	0.79 (0.30–2.07)	0.82 (0.23–2.96)
Overlapping sites (C50.8)	1.85 (1.39–2.45)	1.58 (1.27–1.98)
Unspecified site (C50.9)	2.14 (1.74–2.64)	2.06 (1.59–2.67)
Distant metastases	3.94 (2.72–5.71)	4.70 (3.43–6.45)
Lymph-node metastases	1.26 (1.06–1.48)	1.32 (1.05–1.64)
Congestive heart failure	2.41 (1.83–3.19)	2.25 (1.67–3.03)
Hypertension	1.19 (1.03–1.39)	1.18 (0.98–1.43)
Chronic pulmonary disease	1.14 (0.86–1.51)	1.24 (0.90–1.71)
Diabetes mellitus	1.27 (1.09–1.50)	1.26 (1.03–1.53)
Thyroid disorders	1.04 (0.90–1.20)	1.04 (0.88–1.24)
Chronic kidney disease	1.48 (1.22–1.81)	1.47 (1.17–1.84)
Obesity	1.11 (0.83–1.49)	0.98 (0.67–1.41)
Anaemia	2.41 (1.32–4.40)	2.49 (1.39–4.49)
Depression	1.39 (1.08–1.79)	1.49 (1.13–1.98)

Most associations were stable. Hypertension and obesity were no longer statistically significant after exclusion of this hospital and should be regarded as the least robust of the comorbidity associations.

Table S8. Correction for multiple testing

Benjamini-Hochberg false discovery rate correction applied separately to each of the two primary models. The intercept is excluded; 22 covariates were tested in each model.

Primary model, length of stay (negative binomial)

Variable	RR	Nominal p	FDR-adjusted p	Significant after FDR?
Age 51-60 years	1.02	0.4502	0.4952	No
Age 61-70 years	1.01	0.5856	0.5856	No
Age >70 years	1.14	<0.0001	<0.0001	Yes
Nipple and areola (C50.0)	0.81	<0.0001	<0.0001	Yes
Central portion (C50.1)	1.05	0.0997	0.1290	No
Upper-inner quadrant (C50.2)	0.95	0.0173	0.0260	Yes
Lower-inner quadrant (C50.3)	0.94	0.0178	0.0260	Yes
Lower-outer quadrant (C50.5)	0.94	0.0043	0.0085	Yes
Axillary tail (C50.6)	0.89	0.3837	0.4690	No
Overlapping sites (C50.8)	1.17	0.0003	0.0008	Yes
Unspecified site (C50.9)	1.22	<0.0001	<0.0001	Yes
Distant metastases	1.76	<0.0001	<0.0001	Yes
Lymph-node metastases	1.09	<0.0001	<0.0001	Yes
Congestive heart	1.44	<0.0001	<0.0001	Yes

failure				
Hypertension	1.06	0.0208	0.0285	Yes
Chronic pulmonary disease	1.16	0.0002	0.0006	Yes
Diabetes mellitus	1.08	0.0081	0.0149	Yes
Thyroid disorders	1.02	0.5853	0.5856	No
Chronic kidney disease	1.15	<0.0001	<0.0001	Yes
Obesity	1.04	0.4183	0.4843	No
Anaemia	1.41	0.0014	0.0031	Yes
Depression	1.14	0.0120	0.0202	Yes

16 of 22 associations remained statistically significant after FDR correction.

Primary model, prolonged length of stay (logistic)

**Variable**	**OR**	**Nominal p**	**FDR-adjusted p**	**Significant after FDR?**
Age 51-60 years	1.00	0.9601	0.9853	No
Age 61-70 years	1.00	0.9853	0.9853	No
Age >70 years	1.54	0.0021	0.0075	Yes
Nipple and areola (C50.0)	0.73	0.3016	0.4147	No
Central portion (C50.1)	1.25	0.0595	0.0872	No
Upper-inner quadrant (C50.2)	0.77	0.0043	0.0106	Yes
Lower-inner quadrant (C50.3)	0.81	0.0564	0.0872	No
Lower-outer quadrant (C50.5)	0.80	0.0105	0.0192	Yes
Axillary tail (C50.6)	0.79	0.6302	0.6933	No
Overlapping sites (C50.8)	1.85	<0.0001	0.0001	Yes
Unspecified site (C50.9)	2.14	<0.0001	<0.0001	Yes
Distant metastases	3.94	<0.0001	<0.0001	Yes
Lymph-node metastases	1.26	0.0071	0.0156	Yes
Congestive heart failure	2.41	<0.0001	<0.0001	Yes
Hypertension	1.19	0.0193	0.0326	Yes
Chronic pulmonary disease	1.14	0.3748	0.4850	No
Diabetes mellitus	1.27	0.0031	0.0097	Yes
Thyroid disorders	1.04	0.5928	0.6864	No
Chronic kidney disease	1.48	<0.0001	0.0004	Yes
Obesity	1.11	0.4777	0.5838	No
Anaemia	2.41	0.0043	0.0106	Yes
Depression	1.39	0.0099	0.0192	Yes

13 of 22 associations remained statistically significant after FDR correction.

Table S9. STROBE Statement

This observational study is reported in accordance with the STROBE guideline for cross-sectional studies.

Section/Topic	Item No.	Recommendation	Reported on
Title and abstract	1	Indicate the study's design with a commonly used term in the title or the abstract; provide an informative and balanced summary of what was done and what was found	Title; Abstract
Introduction – Background/rationale	2	Explain the scientific background and rationale for the investigation being reported	Introduction
Introduction – Objectives	3	State specific objectives, including any prespecified hypotheses	Introduction, final paragraph
Methods – Study design	4	Present key elements of study design early in the paper	Section 2
Methods – Setting	5	Describe the setting, locations, and relevant dates, including periods of recruitment, exposure, follow-up, and data collection	Section 2, 2.1
Methods – Participants	6	Give the eligibility criteria, and the sources and methods of selection of participants	Section 2.1
Methods – Variables	7	Clearly define all outcomes, exposures, predictors, potential confounders, and effect modifiers; give diagnostic criteria, if applicable	Section 2.2
Methods – Data sources/measurement	8*	For each variable of interest, give sources of data and details of methods of assessment	Section 2, 2.2
Methods – Bias	9	Describe any efforts to address potential sources of bias	Section 2.3; Discussion, 4.3
Methods – Study size	10	Explain how the study size was arrived at	Section 2.1
Methods – Quantitative variables	11	Explain how quantitative variables were handled in the analyses	Section 2.2, 2.3
Methods – Statistical methods	12	Describe all statistical methods, including clustering, sensitivity analyses, and multiple-testing correction	Section 2.3; Supplement S1–S8
Results – Participants	13*	Report numbers of individuals at each stage of the study	Section 3.1
Results – Descriptive data	14*	Give characteristics of study participants and information on exposures and potential confounders	Section 3.1, Table 1
Results – Outcome data	15*	Report numbers of outcome events or summary measures	Section 3.2, Table 2
Results – Main results	16	Give unadjusted and adjusted estimates and their precision; report category boundaries when continuous variables were categorized	Section 3.3, 3.4; Figures 1–2; Supplement S2–S8
Results – Other analyses	17	Report other analyses done – e.g. analyses of subgroups and sensitivity analyses	Supplement S1–S8
Discussion – Key results	18	Summarise key results with reference to study objectives	Section 4.1
Discussion – Limitations	19	Discuss limitations, taking into account sources of potential bias or imprecision	Section 4.3
Discussion – Interpretation	20	Give a cautious overall	Section 4.4

		interpretation considering objectives, limitations, and multiplicity of analyses	
Discussion – Generalisability	21	Discuss the generalisability of the study results	Section 4.2, 4.3
Other – Funding	22	Give the source of funding and the role of the funders	Funding statement

**Give information separately for exposure categories, where relevant.*
